# The management of slow ovarian response in PCOS patients and its impact on clinical pregnancy outcomes

**DOI:** 10.3389/fendo.2025.1679860

**Published:** 2025-12-02

**Authors:** Yan Zhang, Jihong Yang, Yangbai Li, Xinyue Zhang, Suying Li, Ting Feng, Yun Qian

**Affiliations:** Reproductive Medical Center of the Second Affiliated Hospital of Nanjing Medical University, Nanjing, Jiangsu, China

**Keywords:** controlled ovarian hyperstimulation, polycystic ovary syndrome, slow ovarian response, cumulative clinical pregnancy rate, cumulative live birth rate

## Abstract

**Background:**

The objective of this study was to assess the trade-off between cycle continuation and cancellation in slow ovarian response (SOR) patients, and to evaluate the impact of SOR on embryo developmental potential and clinical pregnancy outcomes.

**Methods:**

A retrospective analysis was performed on 482 patients with PCOS. Patients were stratified into the SOR group (n = 113) and control group (n = 343) in accordance with follicular growth dynamics. Furthermore, data derived from the cycle cancellation group due to SOR (C-SOR group, n = 18) were incorporated for comparative assessment. Clinical outcomes among the respective groups were subsequently subjected to comparative analysis.

**Results:**

The average follicular growth rate in the control group was (1.41 ± 0.45) mm/day, which was significantly higher than that in both the SOR group (1.09 ± 0.55 mm/day, P < 0.05) and the C-SOR group (0.25 ± 0.24 mm/day, P < 0.05). Both SOR and C-SOR groups required significantly more days of Gn and a higher total Gn dose. Notably, supplementation with hCG showed potential for improving ovarian response in patients with SOR. However, if the subsequent follicular growth rate remained below 1.0 mm per day, cycle cancellation was recommended. Although oocytes retrieved was significantly lower in the SOR group than in controls, no intergroup differences were observed in normal fertilization rate, transferable embryo rate, and high-quality embryo rate. Similarly, clinical pregnancy rates after fresh or frozen embryo transfer did not differ between SOR and control groups. However, the SOR group exhibited significantly lower cumulative clinical pregnancy rates (75.22% *vs*. 88.34%, P < 0.05) and cumulative live birth rates (57.52% *vs*. 68.8%, P < 0.05) compared with controls. Logistic regression analysis, after adjustment, revealed that the association between SOR and cumulative live birth rates was not statistically significant (adjusted OR = 0.77, 95% CI: 0.47–1.25, p = 0.29).

**Conclusions:**

Assessment of follicular growth rate in patients with SOR may facilitate clinical decision-making regarding continuation of ovarian stimulation or cycle cancellation. PCOS patients with SOR may benefit from hCG supplementation to enhance ovarian reactivity, thereby facilitating cycle completion and promoting the chance of clinical pregnancy.

## 1 Introduction

Controlled ovarian hyperstimulation (COH) serves as the cornerstone of assisted reproductive technology (ART), with heterogeneity in ovarian response representing a pivotal determinant of treatment outcomes. Traditionally, ovarian responses had been classified into three categories: poor, normal, and high. However, this classification system fails to encompass all clinically relevant scenarios. For instance, there is a subset of women who exhibit normal ovarian reserve but demonstrate suboptimal responses to ovulation induction. For this specific population, the sole use of the term “poor responders” lacks sufficient precision. In previous literature reports, three related terms had been used to describe this phenotype: (1) suboptimal ovarian response, typically defined as retrieval of 4–9 oocytes in patients with adequate ovarian reserve (1–3); (2) slow ovarian response (SOR); and (3) ovarian hypo-response, characterized by delayed follicular development (e.g., no follicles >10 mm on COH days 6-8) (4–6). Notably, the definitions of these terms vary across different literatures and are frequently used interchangeably (7, 8). In the present study, we employed the term “slow ovarian response (SOR)” to define the population, thereby characterizing its phenotypic feature of retarded follicular growth.

Approximately 10–15% of patients developed SOR during COH (7). SOR differs from poor ovarian response (POR) in that it is characterized by normal ovarian reserve, allowing recruitment of sufficient oocytes. However, retarded follicular growth might result in prolonged treatment cycles, increased gonadotropin (Gn) dosage, and heightened economic-psychological burdens for patients. On the other hand, premature treatment termination might miss potential oocyte retrieval opportunities, while whether excessively prolonged stimulation impairs oocyte maturation and embryonic developmental potential remains undefined (9). Some studies had demonstrated an association between prolonged Gn stimulation and adverse pregnancy outcomes (10, 11), whereas others report no correlation or even a negative association (12, 13). This uncertainty underscores the imperative for a systematic review of embryological and clinical outcomes in SOR.

Polycystic ovary syndrome (PCOS) represents the preeminent endocrine disorder, affecting approximately 9–18% of reproductive-aged women (14). The etiology of PCOS remains incompletely understood, with persistent controversies regarding its diagnosis and management. In ART practice, the heterogeneous ovarian response to Gn in PCOS patients constitutes a core determinant of treatment outcomes (15). PCOS patients typically exhibit heightened ovarian reactivity. However, a subset of patients had been overlooked for displaying insufficient sensitivity to exogenous Gn, a phenotype termed SOR. Its optimal management approaches and treatment prognosis remain inadequately defined. SOR may enhance ovarian response through personalized interventions, such as Gn dosage optimization or adjuvant medication administration (1, 16–18). According to published studies, Luteinizing hormone (LH) supplementation had been shown to improve ovarian response in women with SOR during exogenous follicle-stimulating hormone (FSH) stimulation (18–20), which was a beneficial strategy to increase the number of clinical pregnancy rate, implantation rate and retrieved oocytes (6). Human chorionic gonadotropin (hCG) was frequently used as an LH substitute due to its structural and functional homology with LH, as well as its higher binding affinity for LH receptors (21). Adding low-dose hCG to FSH increased mature oocyte yield in long protocol cycles for PCOS patients (22).

This study aimed to explore key clinical questions in PCOS patients with SOR: whether follicular developmental delay could be reversed by hCG supplementation; whether there was a clear threshold for ovarian stimulation duration; and whether prolonged Gn stimulation combined with hCG affected oocyte quality and subsequent embryo development. The objective was to provide evidence-based strategies for the individualized management of SOR.

## 2 Materials and methods

### 2.1 Patients

A retrospective analysis was performed on clinical data of patients in the Reproductive Medicine Centre of the Second Affiliated Hospital of Nanjing Medical University from January 2016 to May 2023. This study was approved by the Reproductive Ethics Committee of the Second Affiliated Hospital of Nanjing Medical University.

Inclusion criteria: (i) IVF cycles; (ii) patients with PCOS.

PCOS was diagnosed based on the Rotterdam criteria (23), requiring the presence of at least two of the following three features:

1. Hyperandrogenism, as confirmed by clinical manifestations or biochemical markers;
2. Ovulatory dysfunction or irregular menstrual cycles;
3. Polycystic ovarian morphology (PCOM) identified via ultrasound, or polycystic ovarian features verified through anti-Müllerian hormone (AMH) level assessment.

Exclusion criteria were as follows: (i) Hyperprolactinemia, thyroid or adrenal disorders, or other endocrine system diseases; (ii) Intrauterine adhesions, uterine space-occupying lesions, or congenital uterine malformations; (iii) Endometriosis; (iv) Chromosomal abnormalities, etc.

**Figure 1:**
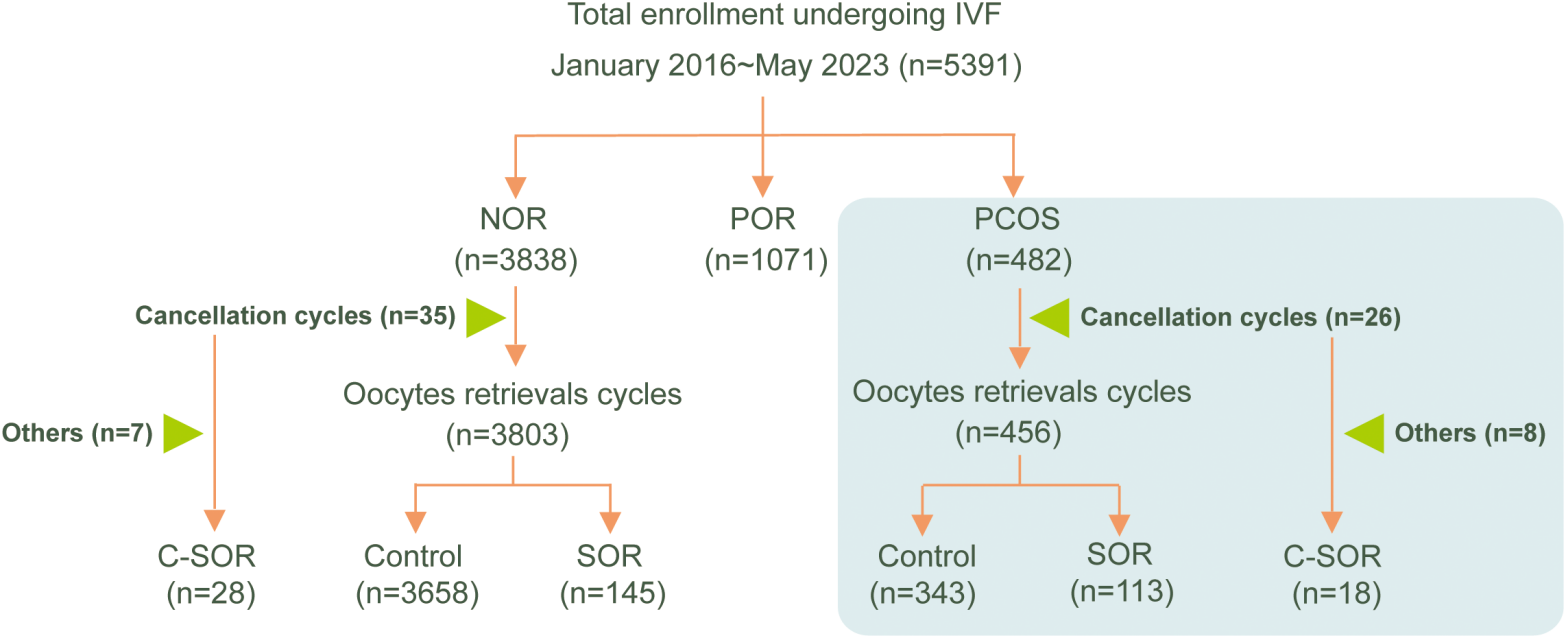
The study flow chart.

The diagnostic criteria for SOR were as follows (4, 8):

1. No follicle development with a diameter > 10 mm on COH days 6–8;
2. Serum estradiol (E2) level < 180 pg/ml on COH days 6–8;

Based on the above diagnostic criteria, patients were categorized into the SOR group (113 cases), the C-SOR group (18 cases, defined as cycles canceled due to SOR), and the control group (343 cases). A flowchart of the patient enrollment process was presented in **Figure 1**.

### 2.2 IVF-ET treatment protocol

#### 2.2.1 Ovarian stimulation protocol

Patients underwent a GnRH agonist long protocol with triptorelin (GnRH-a, 3.75 mg; Lizhu Pharmaceutical, China) administered on days 2–4 of the menstrual cycle. FSH (150–225 U; Lizhu Pharmaceutical, China) was initiated when E2 levels began to rise and at least one follicle reached 5 mm in diameter. Follicular development was monitored by transvaginal ultrasound every 2–3 days. When ≥ 50% of dominant follicles measured > 10 mm, FSH was switched to an equivalent dose of human menopausal gonadotropin (HMG, 150–225 U; Lizhu Pharmaceutical, China). For patients with SOR, follicular stimulation was supplemented with daily hCG (50–100 U; Lizhu Pharmaceutical, China). Ovulation triggering was determined when at least 3 follicles reach ≥ 17 mm or 4 reach ≥ 16 mm in diameter, based on E2, LH, progesterone levels and follicle population size. It was performed via subcutaneous injection of 6,500 U hCG (Lizhu Pharmaceutical, China) at 19:30.

#### 2.2.2 Oocyte collection, fertilization, embryo culture

Thirty-six hours after the trigger, cumulus-oocyte complexes (COCs) were aspirated under transvaginal ultrasound guidance. All cycles underwent IVF insemination. On day 3, transferable embryos were either transferred, cultured to the blastocyst stage, or vitrified for cryopreservation. Embryo quality was assessed daily according to the Istanbul Consensus Workshop on Embryo Assessment (24).

Fresh cycle transfer cancellation was based on four criteria: (i) Progesterone ≥ 1.5 ng/mL on the trigger day; (ii) Embryo-related factors, including no oocytes retrieved, failed normal fertilization, or no embryo transfer (ET), etc.; (iii) Endometrial factors, such as thin endometrium or uterine cavity effusion, etc.; (iv) Miscellaneous factors, including patient discomfort on the scheduled transfer day, partner absence, incomplete documentation, etc.

#### 2.2.3 Embryo transfer and luteal support

One or two embryos were transferred each time via ultrasound-guided intrauterine embryo transfer. For frozen embryo transfer, endometrial preparation was performed using a hormone replacement cycle (HRC). Luteal support was administered as oral progesterone (Abbott, the United States) at 40 mg/day. Biochemical pregnancy was diagnosed 14 days later if urine hCG was positive or blood hCG was ≥ 50 mIU/mL. Clinical pregnancy was defined as the presence of a gestational sac (with or without fetal heart activity) on ultrasound examination 4 weeks after embryo transfer.

### 2.3 Outcome measures

The average follicular growth rate was defined as the daily increment in follicular size following hCG supplementation. The normal fertilization rate (%) was calculated as the ratio of the number of 2PN embryos to the number of oocyte retrieval cycles. The transplantable embryo rate (%) was determined by the ratio of transplantable embryos to the number of 2PN embryos. The high-quality embryo rate (%) was defined as the proportion of high-quality embryos (Grade I and II embryos) to the number of 2PN embryos. The whole embryo freezing rate (%) was determined by the ratio of the number of whole embryos freezing cycles to the number of oocyte retrieval cycles. Clinical pregnancy was defined as the presence of a gestational sac (with or without fetal heart activity) on ultrasound examination 4 weeks after embryo transfer. The cumulative clinical pregnancy rate (%) was calculated as the proportion of clinical pregnancies from all fresh and frozen-thawed embryo transfers following oocyte retrieval. The cumulative live birth rates (%) was defined as the proportion of patients achieving a first live birth from all fresh and frozen-thawed embryo transfers after oocyte retrieval.

### 2.4 Statistical analysis

Statistical analyses were performed using SPSS 26.0 (IBM, Armonk, NY, USA). Continuous data were presented as mean ± standard deviation ( $\bar{\text{x}}$ ± s). The t-test and nonparametric tests (Mann-Whitney U or Kruskal-Wallis) were used for normally and non-normally distributed data, respectively. The chi-square test was employed to compare the rates (%) between groups. Receiver operator characteristic (ROC) curve analysis was conducted to evaluate the follicle growth rate in predicting SOR/C-SOR. The Youden index was used to determine the optimal cut-off value of follicle growth rate for SOR/C-SOR classification. A logistic regression analysis was performed to examine the effect of SOR on the cumulative live birth rates, with BMI and infertility duration included as covariates. P < 0.05 was considered statistically significant.

## 3 Results

### 3.1 Baseline characteristics

Results demonstrated that SOR cycles accounted for 7.04% (304 cases) of all patient cycles. The prevalence of SOR was 4.51% in individuals with normal ovarian response, which contrasted sharply with 27.18% in PCOS patients-a statistically significant difference (P < 0.05, **Supplementary Table 1**). This study included 482 cycles from PCOS patients. Among them, 26 cycles were cancelled before oocyte retrieval, and 18 of these were due to SOR, which is referred to as C-SOR (**Supplementary Table 1** and **Figure 1**). The oocyte retrieval cycle was divided into two groups, including 113 cases of SOR group and 343 cases of normal ovarian response (control) group. The baseline characteristics of the patients were shown in **Table 1**. There were no significant differences between the SOR and control groups in age, FSH, LH, E2, testosterone (T), anti-Müllerian hormone (AMH), or antral follicle count (AFC). Notably, the SOR group had significantly higher BMI and longer infertility duration (P < 0.05).

**Table 1:** Baseline characteristics.

	Control	SOR	C-SOR	95% CI-1	P1	95% CI-2	P2
Number of cycles	343	113	18	NA	NA	NA	NA
Age (years)	29.27 ± 3.90	29.63 ± 3.38	28.44 ± 2.89	-1.12 to 0.37	0.38	-0.49 to 2.86	0.17
BMI (kg/m^2^)	25.11 ± 4.16	28.16 ± 4.69	30.81 ± 4.64	-3.97 to -2.13	0.00*	-5.02 to -0.28	0.03*
Duration of infertility (years)	3.67 ± 2.74	4.29 ± 2.54	3.62 ± 2.06	-1.19 to -0.44	0.04*	-0.58 to 1.92	0.29
Day 2–4 FSH (mIU/ml)	6.31 ± 1.71	6.48 ± 2.49	6.58 ± 1.77	-0.59 to 0.25	0.42	-1.35 to 1.15	0.88
Day 2–4 LH (mIU/ml)	6.23 ± 4.61	6.15 ± 4.89	4.24 ± 1.52	-0.93 to 1.09	0.88	-0.47 to 4.30	0.12
Day 2–4 E2 (pg/ml)	53.42 ± 49.38	44.60 ± 22.76	39.69 ± 15.12	-0.73 to 18.37	0.07	-6.47 to 16.29	0.39
Day 2–4 T (ng/ml)	0.59 ± 0.25	0.58 ± 0.22	0.55 ± 0.23	-0.05 to 0.55	0.96	-0.09 to 0.15	0.62
AMH (ng/ml)	7.70 ± 4.28	7.67 ± 4.43	7.74 ± 2.82	-0.91 to 0.97	0.95	-2.34 to 2.21	0.95
AFC	27.30 ± 7.77	28.66 ± 8.11	32.23 ± 10.34	-3.04 to 0.32	0.11	-7.89 to 0.66	0.10

Data is expressed as mean ± SD, or number (percentage). Independent t-test. Nonparametric test. Chi-squared test. *P < 0.05. NA, not applicable.

SOR, slow ovarian response; C**-**SOR, cancellation cycles by SOR; BMI: body mass index; FSH, follicle stimulating hormone; LH, luteinizing hormone; E2, oestradiol; T, testosterone; AFC, antral follicle count; AMH, anti-Müllerian hormone, CI: confidence intervals.

### 3.2 Follicle growth kinetics

On COH days 6-8, the maximum follicle diameter was 14.8 ± 3.03 mm in the control group, 7.81 ± 1.29 mm in the SOR group, and 7.38 ± 1.08 mm in the C-SOR group. Compared with the control group, the SOR and C-SOR groups exhibited significantly smaller dominant follicle diameters and lower E2 levels (all P < 0.05). The average follicular growth rate in the control group was 1.41 ± 0.45 mm/day, significantly higher than that in the SOR group (1.09 ± 0.55 mm/day, 95% CI: 0.22 to 0.41, P < 0.05). In contrast, the C-SOR group exhibited a significantly lower average growth rate of 0.25 ± 0.24 mm/day. Both the SOR and C-SOR groups required more stimulation days and higher total Gn doses compared with the control group (**Table 2**).

**Table 2:** Comparison of follicle growth status.

	Control	SOR	C-SOR	95% CI-1	P1	95% CI-2	P2
Number of cycles	343	113	18	NA	NA	NA	NA
COH days 6-8							
LH (mIU/ml)	2.85 ± 3.42	2.12 ± 2.11	2.25 ± 1.69	-0.57 to 2.04	0.26	-1.45 to 1.19	0.85
E2 (pg/ml)	1371.2 ± 2165.0	107.8 ± 98.9	60.7 ± 12.7	506.23 to 2060.60	0.00*	2.27 to 118.85	0.04*
Diameter of maximum follicle (mm)							
COH days 6-8	14.8 ± 3.03	7.81 ± 1.29	7.38 ± 1.08	6.19 to 7.78	0.00*	-0.54 to 1.40	0.38
COH days 9-11	19.36 ± 3.36	11.33 ± 3.52	7.92 ± 1.69	6.93 to 8.50	0.00*	2.71 to 6.10	0.00*
COH days 12-14	21.52 ± 3.06	15.04 ± 4.90	8.61 ± 1.27	4.46 to 6.37	0.00*	5.34 to 9.93	0.00*
COH days 15-17	21.73 ± 3.03	19.17 ± 4.63	9.42 ± 1.04	-4.35 to -0.68	0.01*	11.09 to 14.14	0.00*
The average follicular growth rate (mm/day)	1.41 ± 0.45	1.09 ± 0.55	0.25 ± 0.24	0.22 to 0.41	0.00*	0.66 to 1.03	0.00*
Length of stimulation (days)	10.64 ± 2.36	15.70 ± 3.70	16.11 ± 5.11	-5.65 to -4.47	0.00*	-2.40 to 1.57	0.66
Total dose of Gn (IU)	2433.75 ± 711.80	3760.95 ± 1078.84	3637.50 ± 1071.87	-1502.0 to -1152.41	0.00*	-421.95 to 668.85	0.68

Data is expressed as mean ± SD, or number (percentage). Independent t-test. Nonparametric test. Chi-squared test. *P < 0.05. NA, not applicable.

SOR, slow ovarian response; C**-**SOR, cancellation cycles by SOR; LH, luteinizing hormone; E2, oestradiol; COH, controlled ovarian hyperstimulation; Gn, gonadotropin, CI: confidence intervals.

**Figure 2:**
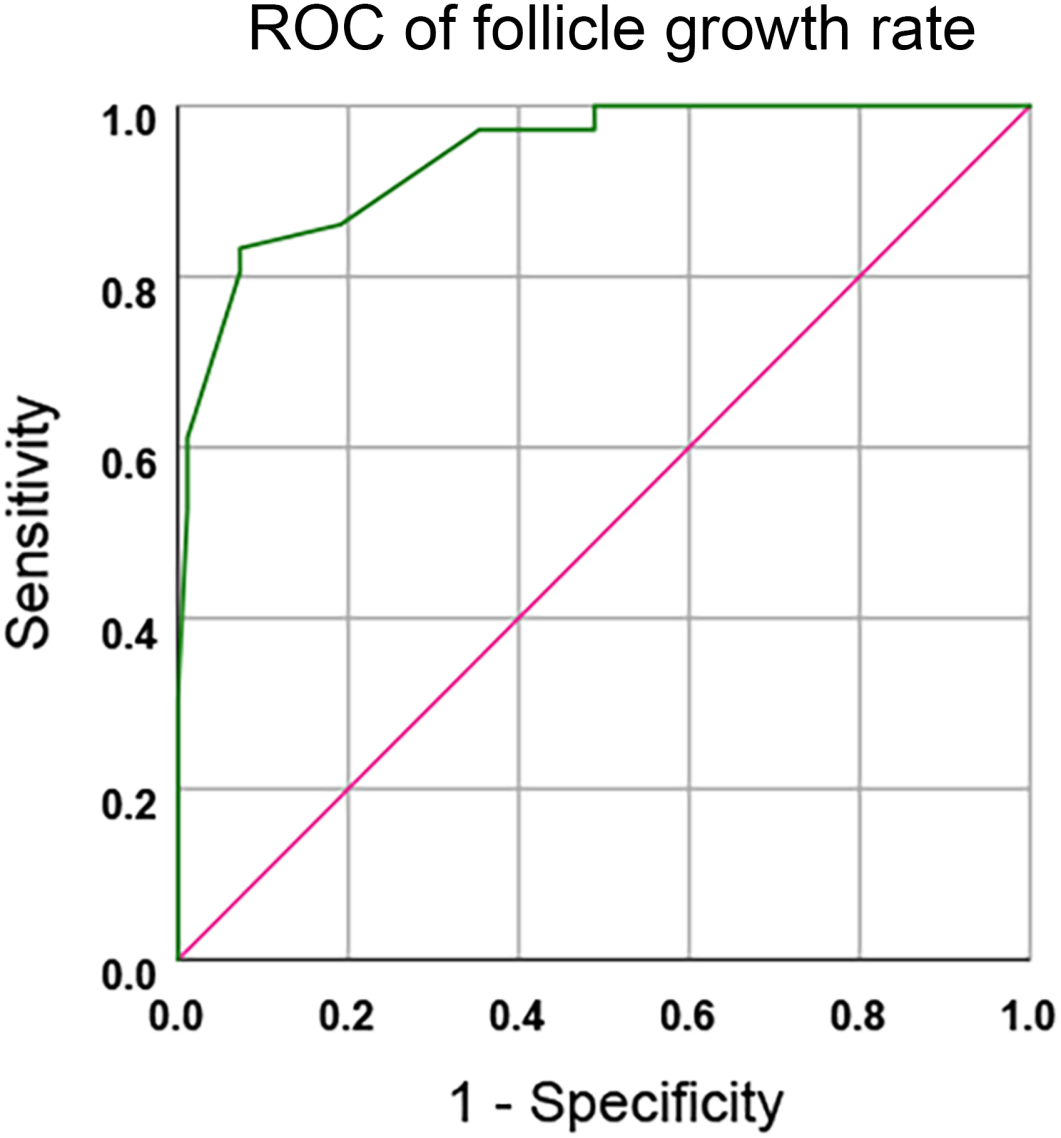
Follicle growth rate in the prediction of SOR/C-SOR by receiver operator characteristic curve (ROC).

**Figure 3:**
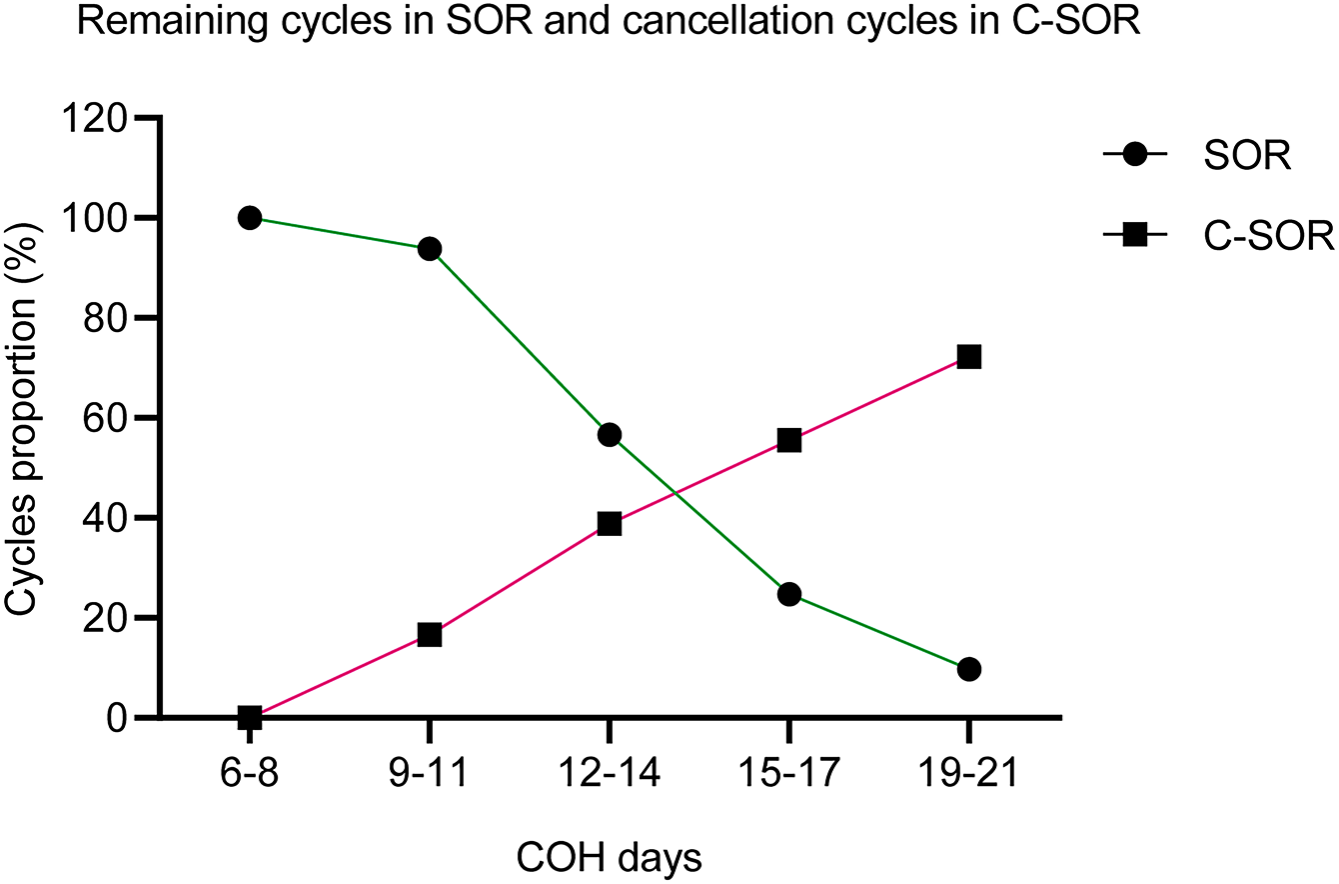
A dual-y-axis line chart displays the trends of remaining cycles after triggering in the SOR group and cancelled cycles in the C-SOR group as COH days increase.

ROC curve analysis revealed a strong correlation between follicular growth rate and SOR/C-SOR classification (AUC = 0.940, P < 0.05; **Figure 2** and **Supplementary Table 2**). The optimal cut-off value of 1.0 mm/day was determined using the Youden index. Biphasic line graph analysis of cycles remaining after triggered in the SOR group and cycles cancelled in the C-SOR group revealed a crossover at approximately days 14.5 of ovarian stimulation (**Figure 3**). This inflection point, specifically COH days 14, may serve as an upper reference limit for Gn in SOR cases. The proportion of SOR and C-SOR cases indicates that 86.3% of slow follicular development cases completed oocyte retrieval cycles with hCG supplementation. These findings suggested PCOS patients may potentially improve ovarian response through supplementation with hCG, thereby completing the oocyte retrieval cycle.

### 3.3 Safety evaluation of the SOR

As presented in **Table 3**, there were no significant differences in E2, progesterone, or LH levels on the trigger day between the SOR and control groups. The number of retrieved oocytes was significantly lower in the SOR group than in the control group (95% CI: 2.39 to 6.96, P < 0.05),. The moderate-to-severe ovarian hyperstimulation syndrome (OHSS) rate did not differ between the SOR and control groups (95% CI: 0.24 to 16.74). No significant differences were observed in normal fertilization rate, transplantable embryo rate, high-quality embryo rate, or total embryo freezing rate between the two groups.

**Table 3:** Clinical outcome comparison.

	Control	SOR	95% CI	P
Number of cycles	343	113	NA	NA
Serum E2 (pg/ml) on the trigger day	5169.51 ± 4536.44	4283.79 ± 4435.96	-87.18 to 1858.62	0.07
Serum LH (mIU/ml) on the trigger day	1.63 ± 2.49	1.70 ± 2.98	-1.21 to 1.32	0.81
Serum P (ng/ml) on the trigger day	1.17 ± 0.92	1.00 ± 0.65	-0.02 to 0.36	0.08
Number of oocytes retrieved	17.83 ± 10.91	15.15 ± 10.02	2.39 to 6.96	0.00*
Moderate to severe OHSS rate (%)	1.75 (6/343)	0.88 (1/113)	0.24 to 16.74	0.52
Normal fertilization rate (%)	71.77 (4094/5704)	71.67 (1007/1405)	0.91 to 1.18	0.62
Transplantable embryo rate (%)	90.82 (3718/4094)	91.06 (917/1007)	0.76 to 1.24	0.81
High-quality embryo rate (%)	61.28 (2509/4094)	63.95 (644/1007)	0.81 to 1.08	0.36
Whole embryo freezing rate (%)	67.64 (232/343)	59.29 (67/113)	0.93 to 2.22	0.11
Fresh embryo transfer cycles	109	46	NA	NA
Clinical pregnancy rate (%)	53.21 (58/109)	54.35 (25/46)	0.48 to 1.91	0.90
Frozen embryo transfer cycles	498	140	NA	NA
Clinical pregnancy rate (%)	49.20 (245/498)	42.86 (60/140)	0.89 to 1.88	0.19
Cumulative Clinical pregnancy rate (%)	88.34 (303/343)	75.22 (85/113)	1.46 to 4.28	0.00*
Cumulative Live birth rate (%)	68.80 (236/343)	57.52 (65/113)	1.05 to 2.52	0.03*

Data is expressed as mean ± SD, or number (percentage). Independent t-test. Nonparametric test. Chi-squared test. P < 0.05. NA, not applicable.

SOR, slow ovarian response; LH, luteinizing hormone; E2, oestradiol; P, progesterone; OHSS, Ovarian hyperstimulation syndrome, CI: confidence intervals.

Normal fertilization rate (%) = number of 2PN/number of oocyte; Transplantable embryo rate (%) = number of transplantable embryos/number of 2PN; High-quality embryo rate = number of high-quality embryos (grade I and II embryos)/number of 2PN; Whole embryo freezing rate (%) = number of whole embryos freezing cycles/number of oocytes retrieved cycles. Clinical pregnancy rate (%) = number of clinical pregnancy cycles/total transplant cycles; Cumulative Clinical pregnancy rate (%) = number of all clinical pregnancy cycles (all fresh and frozen embryo transfer cycles)/number of cycles with oocytes retrievals. Cumulative Live birth rate (%) = number of first live birth cycles (all fresh and frozen embryo transfer cycles)/number of cycles with oocytes retrievals.

In the control group, 109 patients underwent fresh embryo transfer, compared with 49 in the SOR group. Clinical pregnancy rates did not differ significantly between groups (53.21% *vs*. 54.25%, 95% CI: 0.48 to 1.91). For frozen embryo transfer, there were 498 cycles in the control group and 140 cycles in the SOR group, with comparable clinical pregnancy rates (49.2% *vs*. 42.86%,95% CI: 0.89 to 1.88). However, the SOR group showed significantly lower cumulative clinical pregnancy rates (75.22% *vs*. 88.34%, 95% CI: 1.46 to 4.28, P < 0.05) and cumulative live birth rates (57.52% *vs*. 68.8%, 95% CI:1.05 to 2.52, P < 0.05) than the control group.

Logistic regression was performed to adjust for potential confounding effects of BMI and duration of infertility on cumulative live birth rates. After adjusting for these factors, a non-significant trend toward a negative association was observed between SOR and cumulative live birth rates (adjusted OR = 0.77, 95% CI: 0.47–1.25, p = 0.29; **Supplementary Table 3**). These results indicate that SOR was not independently associated with impaired pregnancy outcomes after accounting for BMI and infertility duration.

## 4 Discussion

In this trial, we systematically evaluated multiple key parameters in PCOS patients with follicular growth retardation during COH. The study revealed that BMI was significantly higher in the SOR subgroup, indicating that overweight/obese PCOS patients were more predisposed to SOR. Meanwhile, overweight or obese women tended to require higher doses of gonadotropins (25), and may have reduced ovarian response to ovulation induction drugs (26, 27). The results showed that the SOR group required more stimulation days and a higher total dose of gonadotropins compared with the control group. Follicular growth rates were markedly reduced in the SOR group, reflecting diminished ovarian responsiveness to Gn and implying potential dysregulation in follicular recruitment and maturation. Notably, SOR patients demonstrated reversible ovarian response following hCG supplementation, with a follicular growth rate exceeding 1.0 mm/day. Although clinical pregnancy rates did not differ significantly between the two groups, the SOR group exhibited substantially lower cumulative pregnancy rates and cumulative live birth rates, suggesting potential impairments in embryonic development.

The duration of ovarian stimulation is primarily determined by ovarian response. Multiple indicators reflected ovarian response, including age, basal FSH, basal LH, inhibin B (INB), AFC, AMH, and past history of COH (low or high reaction) (28–31). However, in clinical practice, there are often unpredictable occurrences of SOR. PCOS is a multisystem syndrome with significant physiological and metabolic impacts. Clinical features include hirsutism, infertility, obesity, amenorrhea, insulin resistance, hyperinsulinemia, hypertension, and dyslipidemia (23, 32, 33). PCOS patients with normal baseline parameters may exhibit abnormally slow follicular growth during IVF, in addition to the commonly observed hyper-response. The incidence of SOR in PCOS patients reached 27.18%, which was significantly higher than that in the predicted normal ovarian response group. Clinically, SOR in this population was characterized by “delayed initial response” or follicular growth “stagnation” during ovarian stimulation (34–36). SOR represents a complex, multifactorial outcome that cannot be accurately predicted by single-variable analysis.

The co-administration of hCG during FSH-stimulated cycles may exert a beneficial effect on oocyte maturation (37). hCG supplementation improved follicular development, oocyte retrieval, and the number of transferable embryos (21, 38). hCG supplementation correlated with increased oocyte counts and pregnancy rates (39). In GnRH agonist cycles, adding 100 IU hCG to FSH improved ovarian stimulation response in SOR patients (40). Low-dose hCG also did not affect endometrial development (41). Results indicated that 27.18% of PCOS patients developed SOR during long protocol IVF cycles, and 86.3% of these cases yielded mature oocytes following hCG supplementation. Consistent with our findings, SOR patients could recruit sufficient oocytes despite increased gonadotropin consumption (16). Laboratory outcomes and clinical pregnancy rates (in both fresh and frozen cycles) were comparable between the SOR and control groups. This suggests that the timely identification of SOR, followed by appropriate interventions, may be effective in normalizing ovarian response (8, 16).

However, both cumulative pregnancy and live birth rates were significantly lower in the SOR group than in the control group, indicating that slow ovarian response may affect pregnancy outcomes. The reduction may be attributed to: 1) higher BMI in the SOR group. In PCOS women, BMI was positively correlated with total gonadotropin dosage and stimulation duration, and served as a risk factor for ovarian adverse reactions and poor response (42). Patients with higher BMI yielded fewer oocytes, fewer transferable embryos, and lower fertilization and pregnancy rates (43). 2) the number of retrieved oocytes decreased in SOR group. Higher oocyte counts were positively associated with increased cumulative pregnancy and live birth rates (44–46). 3) previous studies have suggested potential mechanisms: prolonged administration of hCG may compromise endometrial receptivity (47), prolonged gonadotropin stimulation was associated with reduced quality of transferable embryos (48, 49), and SOR had been linked to suboptimal pregnancy outcomes (8). Despite inconsistent findings in the literature, exposure to high-dose gonadotropins may induce chromosomal abnormalities in oocytes and increase aneuploidy rates in granulosa cells (50), and significantly increase the incidence of complications like thromboembolism (51). Thus, regulating the duration of gonadotropin stimulation is essential.

On the other hand, logistic regression analysis, after adjustment for BMI and infertility duration, revealed no statistically significant association between SOR and cumulative live birth rates (p = 0.29). Neither BMI (p = 0.20) nor infertility duration (p = 0.86) were significant predictors in the model. These non-significant findings could point to complex interdependencies among these variables or may reflect the limited statistical power of the present study to identify independent effects. Therefore, future studies with larger sample sizes are warranted to validate the relationships among these variables and clarify their independent roles.

We further analyzed the second cycle of C-SOR patients, in which ovarian stimulation protocol was modified and/or the starting dose was increased to 300 IU to prevent SOR. However, the results showed that among 18 patients, 5 remained C-SOR (27.8%), 4 developed SOR (22.2%), and 9 exhibited normal ovarian response (**Supplementary Table 4**). Except for C-SOR cycles, the duration of Gn days was 12 ± 2.89 days and a total dosage of Gn was 2994 ± 816.5 U. Although both Gn duration and total dosage in the second cycle of the C-SOR group were significantly lower than those in the first-cycle SOR group, the cumulative values of the two cycles increased substantially. The time-saving advantage compared to repeated IVF cycles was undeniable. Thus, early identification of SOR and proactive optimization of ovarian response yielded substantial benefits.

This study had several limitations that warrant acknowledgment. First, as a retrospective cohort study, the analysis was limited by its sample size and the imbalance in group sizes. Although selection bias was minimized by focusing on GnRH-a protocol cycles in PCOS patients, findings require validation through large-sample randomized controlled trials. Second, as a clinical observational study, the present investigation aimed to identify and optimize SOR; it did not, however, include fundamental investigations into ovarian sensitivity. Future studies should further elucidate the pathogenic pathways of SOR and its mechanisms underlying pregnancy outcomes. Third, the small sample size of the C-SOR subgroup which comprised only 18 cases, limits the statistical power of this study. Consequently, the precision of the proposed upper time limit (COH days 14) was constrained, and this parameter should currently be regarded as a preliminary reference in clinical practice. Further validation with larger clinical datasets is required to substantiate these findings.

In summary, this study demonstrated that for PCOS patients with SOR, the decision to continue follicular monitoring was based on whether the follicular growth rate reached 1 mm/day. It was found that hCG supplementation may potentially improve ovarian response and SOR did not independently compromise pregnancy outcomes.

## Data Availability

The original contributions presented in the study are included in the article/**Supplementary Material**. Further inquiries can be directed to the corresponding author.
