# Supplementary material for: The management of slow ovarian response in PCOS patients and its impact on clinical pregnancy outcomes
Source: Front Endocrinol (Lausanne). 2025 Dec 2;16:1679860. doi: 10.3389/fendo.2025.1679860 (PMC12705353; doi:10.3389/fendo.2025.1679860)
Supplement: Supplementary file 1 [file Table1.docx]

**Supplementary Table 1 Proportion of SOR in PCOS and NOR**

|  | p-NOR | p-PCOS | P |
| --- | --- | --- | --- |
| Number of cycles | 3838 | 482 | NA |
| Cancellation cycles rate (%) | 0.91 (35/3838) | 5.39 (26/482) | 0.00* |
| Cancellation cycles rate due to premature follicular ovulation, etc. (%) | 0.00 (7/3838) | 1.66 (8/482) | 0.00* |
| C-SOR (%) | 0.00 (28/3838) | 3.73 (18/482) | 0.00* |
| Number of cycles with oocytes retrievals | 3803 | 456 | NA |
| Control (%) | 95.31 (3658/3838) | 71.16 (343/482) | 0.00* |
| SOR (%) | 3.78 (145/3838) | 23.44 (113/482) | 0.00* |
| SOR + C-SOR (%) | 4.51 (173/3838) | 27.18 (131/482) | 0.00* |

Data is expressed as mean ± SD, or number (percentage). Independent t-test. Nonparametric test. Chi-squared test. * P < 0.05. NA: not applicable.

NOR, normal ovarian response; PCOS, Polycystic ovary syndrome; SOR, slow ovarian response; C**-**SOR, cancellation cycles due to SOR.

**Supplementary Table 2 ROC curve for predicting SOR/C-SOR by follicle growth rate**

| Variable | AUC | SE | P-value | 95% CI |
| --- | --- | --- | --- | --- |
| Follicular growth rate | 0.940 | 0.020 | 0.000 | 0.900-0.980 |

**Supplementary Table 3 Logistic regression analysis was performed to examine the effect of SOR on the cumulative live birth rates, with BMI and infertility duration included as covariates.**

|  | P-value | Exp (B) | 95% CI | |
| --- | --- | --- | --- | --- |
|  |  |  | **Lower limit** | **Upper limit** |
| SOR | 0.289 | 0.767 | 0.470 | 1.251 |
| BMI | 0.202 | 0.969 | 0.922 | 1.017 |
| Duration of infertility | 0.857 | 0.993 | 0.918 | 1.074 |

SOR, slow ovarian response; BMI, body mass index.

**Supplementary Table 4 Characteristics of the second oocyte retrieval cycle of C-SOR**

| C-SOR (27.8%) | Short protocol: 1 case  Micro-stimulation protocol: 4 cases |
| --- | --- |
| SOR (22.2%) | Short protocol: 1 case  Micro-stimulation protocol: 3 cases |
| NOR (50%) | Antagonist protocol: 1 case  Short protocol: 2 cases  Micro-stimulation protocol: 5 cases  Long protocol: 1 case |

NOR, normal ovarian response; SOR, slow ovarian response; C**-**SOR, cancellation cycles due to SOR.
